# Relationship of epilepsy on the linguistic-cognitive profile of children with ASD: A systematic review of the literature

**DOI:** 10.3389/fpsyg.2023.1101535

**Published:** 2023-03-29

**Authors:** Alejandro Cano-Villagrasa, Francisco José Moya-Faz, Miguel López-Zamora

**Affiliations:** ^1^Health Sciences PhD Program, Universidad Católica de Murcia UCAM, Campus de los Jerónimos, Murcia, Spain; ^2^Facultad de Ciencias de la Salud, Valencian International University, Valencia, Spain; ^3^Facultad de Ciencias de la Salud, Universidad Católica San Antonio de Murcia (UCAM), Murcia, Spain; ^4^Departamento de Psicología Evolutiva y de la Educación, Facultad de Psicología y Logopedia, Universidad de Málaga, Málaga, Spain

**Keywords:** autism spectrum disorder, epilepsy, cognition, language, comparison

## Abstract

**Introduction:**

The prevalence of comorbidity between epilepsy and Autism Spectrum Disorder (ASD) in the pediatric age increased significantly in recent years. The onset of epilepsy negatively influences the abilities of the user with ASD. Thus, epilepsy will be a disabling factor that will reduce the cognitive-linguistic skills of users with ASD. The main objective of this work is to review the current scientific literature and to compare the relationship of epilepsy on the development of cognitive and linguistic skills of children with ASD.

**Methods:**

In this regard, a systematic search was carried out in the main sources (Medline, PubMed, WOS, ResearchGate and Google Scholar). 481 articles were identified, from which, after meeting the different inclusion and exclusion criteria, a total of 18 studies of relevance to the objectives of this work were selected.

**Results:**

The results reflect that, at a global level, epilepsy significantly influences the performance of cognitive- linguistic skills in people with ASD.

**Discussion:**

In conclusion, epilepsy in the ASD population leads to a reduction in cognitive and linguistic abilities, which respond to the different types of epilepsy and their location, significantly impacting the quality of life and basic activities of daily living of the user with ASD.

## Introduction

The study of comorbidity between epilepsy and Autism Spectrum Disorder (ASD) has acquired importance in recent years due to a significant increase in the prevalence found in the profiles of users with these two comorbid pathologies (Spence and Schneider, 2009; Mannion and Leader, 2014a; Strasser et al., 2018). On the one hand, epilepsy is defined as a brain alteration characterized by a permanent predisposition to generate seizures and by the neurobiological, cognitive, psychological, and social consequences derived from this condition (Pastorino et al., 2021). Its diagnosis is possible after the user experiences an isolated seizure which is not caused by any external factor (Muñoz Yunta et al., 2006). Epilepsy encompasses a series of signs and symptoms of various etiologies that are grouped according to the type of seizures, electroencephalogram (EEG) alterations, age of symptom onset, the existence of precipitating factors, cause, prognosis, response to treatment, and anatomical location of the initial focus of the seizures (Ewen et al., 2019). On the other hand, ASD is classified within neurodevelopmental disorders. The most plausible hypothesis with the most scientific support places its origin in a complex and multifactorial genetic predisposition that produces neuronal abnormalities due to alterations in early synaptic plasticity during development (Poduri and Lowenstein, 2011; Tuchman and Cuccaro, 2011). These children could have common mechanisms due to neurobehavioral phenotypes that, interacting with environmental factors, would produce significant deficits in expressive and receptive language dimensions (Miranda et al., 2022). Without considering the etiological variability of these disorders, their different development according to age and the modification of the evolutionary course of the patient that these disorders impose separately, when both disorders appear in an individual, the impact produced is difficult to detect and quantify. Consequently, this comorbidity is significantly changing the way in which ASD is evaluated and intervened.

In this regard, an increase in the incidence of clinical epilepsy and/or epileptiform activity on EEG in children with ASD is being observed, possibly due to the sensitization of clinical protocols to this comorbidity. However, studies are far from homogeneous, so the association between these two disorders can be estimated at a percentage ranging from 7 to 42% depending on the research (Ghacibeh and Fields, 2015). This variability may occur due to the clinical differences of both disorders and their evolutionary course. For example, in ASD, seizures can begin at any age; however, a higher rate of occurrence of these episodes is observed in early childhood and early adolescence. In addition to this, it is estimated that 23% of children with this comorbidity also have hyperactivity, so other symptoms that could be present and complicate the differential diagnosis must be added. Another reason could be due to differences in the distribution of the manifestations of ASD in current studies, where unequal importance is given to the individual characteristics of the users, together with the form of onset and typology of epilepsy (Besag, 2015; Dunn et al., 2016).

The study of comorbidity between ASD and epilepsy is not recent. In the first clinical descriptions of ASD, Kanner (1943) indicated that, in patients with autism, episodes of epileptic seizures were already observed. According to him, these episodes could have repercussions on a worse acquisition of linguistic and social competences in children with both disorders (Rutter, 1978). Nowadays, neurologists specializing in childhood epilepsy carry out an extensive evaluation to determine the existence of this comorbidity, since early detection is a good prognostic factor for the development of cognitive-linguistic and social skills of users with epilepsy and ASD, especially if the disease manifests itself in early childhood (Muñoz-Yunta et al., 2008). This way, it is common to incorporate families in the evaluation process so that they are the ones who detect the possible existence of epileptic seizure episodes (Auvin, 2021).

It should be highlighted that, in clinical practice, there are difficulties in making a differential diagnosis of ASD and epilepsy in relation to other diseases. Generally, the involution in the maturational processes of the child is taken as a reference, in addition to alterations in EEG patterns, but these symptoms are common to other pathologies that may interfere with the diagnosis of ASD. Among them, Landau-Kleffner Syndrome (LKS), also known as epileptic aphasia, stands out. During the early stages of this syndrome, prolonged spike-wave activity during sleep is common, which may cause a conflict in establishing an accurate diagnosis (Pablo et al., 2002). EEG changes in the infantile population with LKS are characterized, as a rule, by pronounced paroxysmal activity. Likewise, spike-wave complexes and multiple sharp waves with prevalence in the temporal regions with a unilateral or bilateral profile and generally asymmetrical are detected. Thus, in most cases cognitive and linguistic disturbances develop. However, in studies such as that of Berkvens et al. (2015), the anomalous epileptiform activity is localized is in temporal areas (30%), central areas (28%), frontal areas (23%) and occipital areas (8%). It is also common in LKS for there to be a rapid deterioration in expressive language comprehension (Matthews et al., 2012; El Achkar and Spence, 2015) due to the involvement of the respective brain areas, which can be confused with the regression of language and cognitive skills that occurs in childhood in children with ASD.

Regarding the influence of epilepsy during the critical periods of childhood development and how it can affect its differential diagnosis, the need for an exhaustive and early analysis is highlighted, since this disorder has a direct impact on the development of higher cognitive skills, including language (Junior et al., 2021). This can cause epilepsy to be confused with other pathologies. This is because brain regions mature earlier than others in chronological order. Therefore, if this development is disrupted by the onset of epilepsy in key areas for cognition and language, such as the Sylvian fissure, the temporal area, and the insula, all this will negatively affect the development of these processes (Bitton et al., 2015; Trauner, 2015). In fact, seizure episodes may occur without the patient presenting any clinical symptomatology, which may affect the overall maturation process, but further hindering the diagnosis (Karrasch et al., 2017). In summary, these symptoms can interfere with the correct diagnosis of epilepsy.

Regarding the comorbidity of both disorders, it has been reported that between 10 and 15% of children with ASD experience a regression in acquired behaviors after the initial stage of maturational development, especially in language skills, social interaction, and symbolic play (Matthews et al., 2012). In the last two decades, studies related to neurodevelopmental disorders have questioned whether epilepsy would play a role in the onset of cognitive and language disorders (Bitton et al., 2015). Currently, it is considered that epilepsy can mainly influence cognition and behavior at a very early age, even in cases where there are no clinically visible seizures (Tuchman et al., 2013; Galán-López et al., 2017). This has led to an increase in research to find evidence of subclinical epilepsy in children with developmental problems (Smith, 2016), one of the most relevant being ASD due to its prevalence and clinical interest. On the other hand, epilepsy is also related to language disorders. In the process of language development (both expressive and receptive), epilepsy can negatively condition its acquisition, as well as favor its degradation. When language has been acquired in a normal chronological order, neurodevelopmental disorders such as ASD that co-occur with epilepsy may present significant language impairment or delay (Berg, 2016). In these cases, the influence of epilepsy on ASD symptoms should be considered, since the development of higher psychological processes is related to communication and language. Therefore, continued exposure to epileptic seizures could impair aspects such as empathy and activities where expressive language takes on greater weight, hindering the child's communication with the surrounding environment (Braakman et al., 2012).

Within this framework, the main objective of this systematic review of the literature is to analyze the co-existence and relationship of epilepsy in the deterioration of cognitive-linguistic skills of those users who present an ASD in childhood. Likewise, this work establishes a series of specific objectives: (I) to analyze the general symptomatology of children presenting an ASD with epilepsy, and (II) to explore the existing differences between both sexes in ASD children with epilepsy.

## Materials and methods

In the present article, a systematic review of the literature regarding ASD and epilepsy is presented. To ensure the accuracy of the study's question, we specified the criteria related to users with ASD and epilepsy, comparing the symptoms and the results of their respective diagnostic evaluations (Liberati et al., 2009). The question to be answered is what relationship epilepsy has on the development of cognitive-linguistic skills of users with ASD, following the criteria of the following PICO model: (P) People diagnosed with ASD and epilepsy. (I) Does not apply. (C) Does not apply. (O) Decrease in cognitive-linguistic skills. On the other hand, this literature review has followed the guidelines and recommendations established in the Preferred Reporting Items for Systematic Reviews and Meta-Analyses statement (PRISMA; Yepes-Nuñez et al., 2021).

### Search strategy

An electronic search was carried out between November 2021 and June 2022 in the following databases: Medline, Pubmed, PsycINFO, Web of Science and Google Scholar. The search was restricted to peer-reviewed articles published in journals and/or available online and written in Spanish or English. To ensure that the included articles were current, the search was restricted to the last 11 years. In the study search, the following terms were considered in the article title, abstract and key words: epilepsy, ASD, and linguistic-cognitive development.

As for the search strategy, the following terms were used, combined with Boolean operators: “(epilepsy relationship) and (children with ASD) and (linguistic development)” “(epilepsy comorbidity) and (children with ASD) and (cognitive development)” “(epilepsy co-existence) and (children with ASD)” “(cognitive and linguistic development) and (children with ASD and epilepsy)” “(epilepsy) and (ASD) and (linguistic and cognitive skills).”

### Study selection process

To obtain highly relevant published studies based on the study's objectives and the PRISMA guidelines (Page et al., 2021), inclusion and exclusion criteria were established prior to the systematic search. To improve the quality of the search, a peer review was carried out using the standardized Peer Review of Electronic Search Strategies (PRESS) instrument (Sampson et al., 2009). In this manner, in the first study pre-selection stage, based on the information provided in the title and abstract, the following inclusion criteria were considered for the selection of articles of relevance: (I) the samples of the studies reflect the existences between the comorbidities of the two studied conditions (epilepsy-ASD and cognitive competence or language competence); (II) these conditions have been specifically assessed; and (III) the assessments have been carried out by professionals in psychology or medicine. Regarding exclusion criteria, the following were considered: (I) single case-reports, without a methodological design; (II) the study of epilepsy unrelated to ASD or vice versa; and (III) review papers, editorials, or abstracts of studies presented at congresses.

In the second stage of study selection, the complete analysis of the articles that met the indicated inclusion criteria was performed. In this process, the following inclusion criteria were considered: (I) the study participants had an official diagnosis of ASD; (II) the article precisely described the parameters of the assessment performed in the participants; and (III) the individual characteristics of the linguistic or cognitive performance of the study participants were specified.

The selection process of the studies included in this systematic review was carried out by two researchers and consisted of 4 phases: In the first phase, we proceeded to review the scientific literature in the main databases in the field of psychology and medicine. In the second phase, each of the researchers checked the title and abstract of each eligible article, according to the previously established inclusion and exclusion criteria. In the third phase, the articles that met the inclusion and exclusion criteria were analyzed in detail by full-text screening. Finally, in the fourth phase, we concluded with the scrutiny and discarding of the articles that did not address the topic of interest of this systematic review, evaluating the main information of the selected articles.

### Final study selection

**Figure 1:**
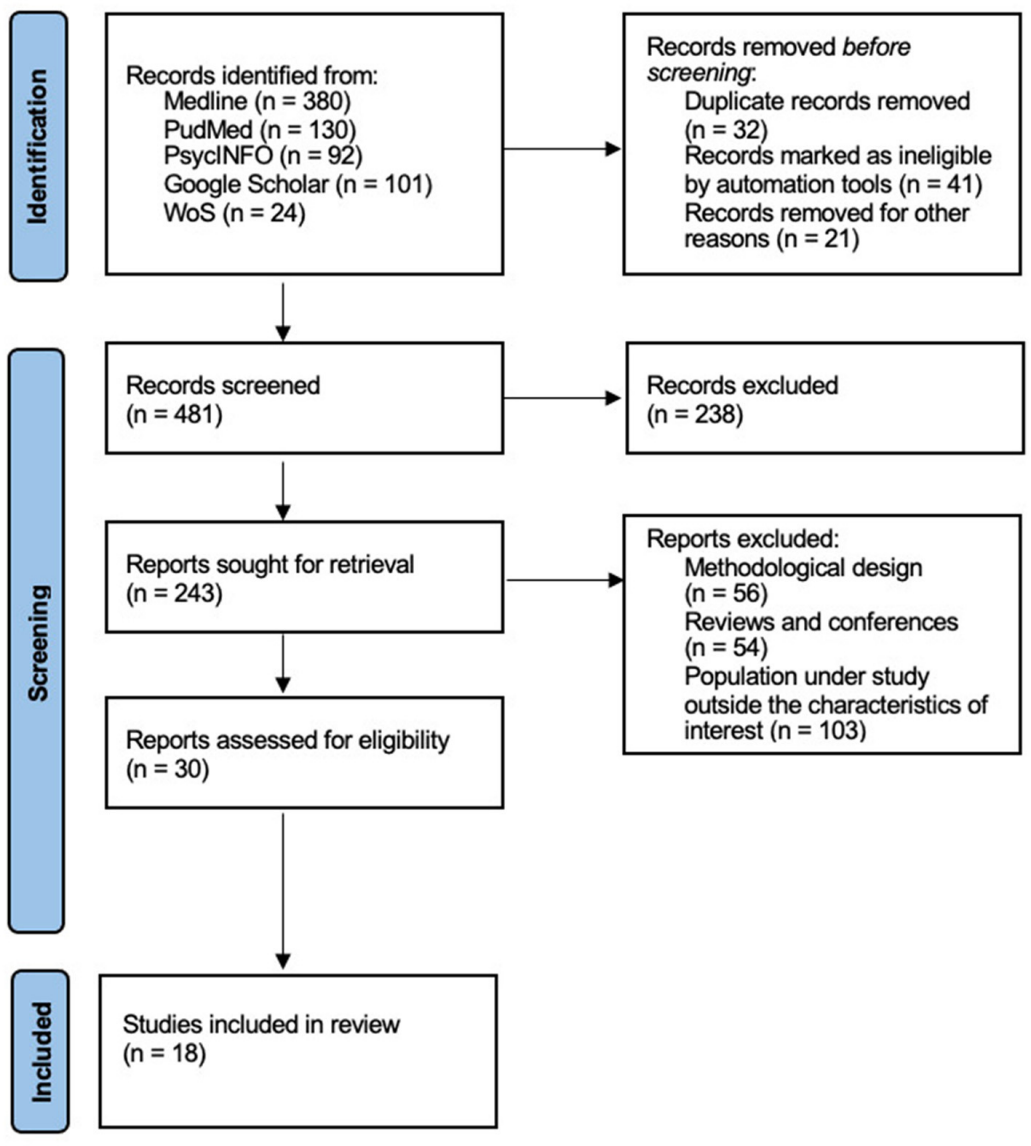
Flow chart of study selection.

During the initial search for studies, a total of 481 articles that were potentially eligible were found, which were distributed as follows: 134 from Medline, 130 from Pubmed, 92 from PsycINFO, 101 from Google Scholar (through a manual bibliographic search) and 24 from Web of Science. Figure 1 shows a flow chart illustrating the study selection process, considering the PRISMA statement (Moher et al., 2009). From this total, 238 duplicated references were discarded.

Thus, based on the information contained in the title and abstract sections of the manuscript, 243 articles were identified as potentially eligible for review. From the 243 eligible articles, 213 were discarded because they did not meet the previously established inclusion and exclusion criteria: (I) 103 did not include children with ASD or epilepsy, (II) 56 articles were single case reports without an adequate methodological design, and (III) 54 were reviews and papers presented at research congresses.

Of the remaining 30 articles, a detailed assessment of each was carried out, of which 12 were rejected because they did not meet the previously established selection criteria, leaving a total of 18 articles that were finally included in this systematic review.

### Study description

The methodological characteristics of the selected articles are summarized in Table 1. The sample size of the selected articles ranged from 20 to 85,248 participants (*M* = 5422). The studies used different community services, health systems or a combination of other processes to select their final sample. Regarding the ages of the subjects in the studies, it is observed that this was between 2 and 18 years old.

**Table 1:** Description of study selected.

**Authors (year)**	**Title**	**Objective**	**Results**	**Conclusions**	**N**	**Age (SD)**	**Journal (IF)**
**Characteristics and general symptomatology**							
Reilly et al. (2015)	Features of autism spectrum disorder (ASD) in childhood epilepsy: a population-based study.	To describe neurobehavioral comorbidity in children with both epilepsy and ASD, to identify the distribution of features of ASD in children with epilepsy, and to assess the utility of the ASSQ as a screening tool for ASD in children with epilepsy.	Parents reported significantly (*P* < 0.05) more features of ASD on the ASSQ compared with teachers. Factors significantly associated with responses on the ASSQ included respondent (parents reported more features), school placement (more features in specialized settings), and respondent by school placement interaction.	Effective screening for ASD in children with epilepsy will need a consideration of the impact of informant and school placement on ratings.	117	5–15 (2.16)	Epilepsy Behav (2.96)
Berg et al. (2011)	Risk and correlates of autism spectrum disorder in children with epilepsy: a community-based study.	To estimate the prevalence of ASD in a representative, prospectively identified, community-based cohort of children with epilepsy and provide information about the main factors associated with ASD in this context.	West syndrome (prevalence ratio = 4.53, *P =* 0.002), and intellectual impairment (prevalence ratio = 4.34, *P =* 0.002), were independently associated with autism spectrum disorder. Absent West syndrome, male gender was associated with autism spectrum disorder (prevalence ratio = 3.71, *P =* 0.02).	The most important determinants of autism spectrum disorder in the general population (intellectual impairment and male sex) are also important in young people with epilepsy.	613	3.5–10 (6.72)	Journal of child neurology (2.36)
Sathyabama (2019)	Clinical characteristics and demographic profile of children with Autism Spectrum Disorder (ASD) at child development clinic (CDC), Penang Hospital, Malaysia.	To explore socio-demographics and clinical characteristics of children with Autism Spectrum Disorder (ASD) at Child Development Clinic (CDC), Penang Hospital.	History of speech regression was noted in 14.8%, epilepsy and genetic disorders in 9.4% and 5.7% respectively. Sleep problems was reported in 29.3%, dietary issues 22.1%, challenging behavior 24.2% and ADHD 14.2%. Mean age of the father and mother at birth was 33.6 and 31.6 years respectively.	Report a higher male to female ratio and mean age at referral with some similar rates of neurodevelopmental and medical comorbidities and relatively younger parental age with higher parental education levels.	331	5.6 (3.6)	The Medical journal of Malaysia (0.53)
Jokiranta et al. (2014)	Epilepsy among children and adolescents with autism spectrum disorders: a population-based study.	To examine associations between epilepsy and autism spectrum disorders (ASD).	Epilepsy was associated with ASD regardless of the subgroup after adjusting for covariates. The associations were stronger among cases with intellectual disability, especially among females.	Their findings emphasize the importance of examining neurodevelopmental pathways in ASDs, epilepsy, and intellectual disability.	4,705	5.5–9.3 (3.3)	Journal of autism and developmental disorders (4.34)
Sharma et al. (2021)	Epilepsy and EEG Abnormalities in Children with Autism Spectrum Disorders.	To evaluate the prevalence of epilepsy and electroencephalographic abnormalities in children with autism spectrum disorders (ASD) and determine their risk factors.	Epilepsy was reported in 23% and subclinical electroencephalographic abnormalities were documented in 8%. The most common seizure types were generalized-onset tonic-clonic (48%), focal-onset with impaired awareness (17%), and focal to bilateral tonic-clonic seizures (17%). In children with subclinical epileptiform discharges, focal abnormalities were most common (75%) and were maximally seen over the temporal region (50%). Subnormal intellect (88.6%) and abnormal global developmental score (82%) were noted in the majority of children.	Epilepsy is seen in up to one-fourth children with ASD. Female gender and adverse perinatal events are independent risk factors for epilepsy. Subclinical or isolated EEG abnormalities are associated with abnormal neurological examination.	100	3–14 (5.6)	Indian Journal Pediatric (5,31)
Norrelgen et al. (2015)	Children with autism spectrum disorders who do not develop phrase speech in the preschool years.	To examine this ratio in a population-based community sample of children.	The proportion of children who met the criteria for non-verbal, minimally verbal, and phrase speech were 15, 10, and 75%, respectively.	The single most important factor linked to expressive language was the child's cognitive level, and all children classified as being non-verbal or minimally verbal had intellectual disability.	165	4–6 (2)	Autism (5.68)
Celik et al. (2021)	Evaluation of the clinical characteristics of children with autism spectrum disorder and epilepsy and the perception of their parents on quality of life.	To examine the clinical conditions that mediate this comorbidity, compare parental quality of life in isolated ASD and ASD with epilepsy, demonstrate the relationships between clinical and EEG findings obtained in diagnostic evaluation, and examine the results in light of the literature.	Mean (SD) total scores in the Quality of Life in Autism Questionnaire were 131.84 (10.68) among mothers of children with ASD-epilepsy and 148.33 (14.03) among mothers of children with ASD alone (*P* < 0.001).	Many psychiatric and medical conditions can co-occur with ASD. Determining the prognostic criteria for ASD is of great importance in coordinating lifelong autism rehabilitation. Improving autism-specific symptoms will benefit children with ASD as well as help mitigate parental anxiety.	154	3.5–16.3 (3.16)	Epilepsy research (2.75)
**Gender differences**							
Amiet et al. (2013)	Does epilepsy in multiplex autism pedigrees define a different subgroup in terms of clinical characteristics and genetic risk?	The aim of this study was to assess: 1. The prevalence of epilepsy in multiplex autism and its association with genetic and non-genetic risk factors of major effect, intellectual disability and gender. 2. Whether autism and epilepsy cosegregate within multiplex autism families	The prevalence of epilepsy was 12.8% in cases with ASD and 2.2% in siblings without ASD (*P* < 10–5). With each RCPM or VABS measure, the risk of epilepsy in multiplex autism was significantly associated with intellectual disability, but not with gender. Identified risk factors (genetic or non-genetic) of autism tended to be significantly associated with epilepsy (*P =* 0.052). When children with prematurity, pre- or perinatal insult, or cerebral palsy were excluded, a genetic risk factor was reported for 6/59 (10.2%) of children with epilepsy and 12/395 (3.0%) of children without epilepsy (*P =* 0.002).	Epilepsy in multiplex autism may define a different subgroup in terms of clinical characteristics and genetic risk.	664	9.2 (5.02)	Molecular autism (6.26)
Thomas et al. (2017)	Brief Report: Prevalence of Co-occurring Epilepsy and Autism Spectrum Disorder: The U.S. National Survey of Children's Health 2011-2012.	To quantify the cooccurrence of epilepsy and ASD in a large nationally representative sample of the most recent survey of the NSCH 2011–2012 and examine child characteristics that are associated with this co-occurrence.	In the overall analytic sample of 85,248 children ages 2–17, there were 1,604 children with ASD (prevalence of 1.8 %) and 1,083 children with epilepsy (prevalence of 1.2 %). Epilepsy was reported to co-occur in 8.6 % of ASD cases.	In children with ASD, the co-occurrence of epilepsy was associated with increasing child age, female gender, intellectual disability, speech problems and lower socioeconomic status.	85,248	2–17 (2.23)	Journal of autism and developmental disorders (5.25)
Viscidi et al. (2013)	Clinical characteristics of children with autism spectrum disorder and co-occurring epilepsy.	To estimate the prevalence of epilepsy in children with Autism Spectrum Disorder (ASD) and to determine the demographic and clinical characteristics of children with ASD and epilepsy in a large patient population.	The average prevalence of epilepsy in children with ASD 2–17 years was 12.5%; among children aged 13 years and older, 26% had epilepsy. Epilepsy was associated with older age, lower cognitive ability, poorer adaptive and language functioning, a history of developmental regression and more severe ASD symptoms	This is among the largest studies to date of patients with ASD and co-occurring epilepsy. Based on a representative sample of children with ASD, the average prevalence of epilepsy is approximately 12% and reaches 26% by adolescence. Independent associations were found between epilepsy and older age and lower cognitive ability.	5,815	13–17 (3.12)	PLoS ONE (3.75)
Save-Pédebos et al. (2016)	The development of pragmatic skills in children after hemispherectomy: contribution from left and right hemispheres.	To analyze whether any correlation exists with age at surgery and side of surgery on pragmatic skills following H.	The whole group had significant deficits in all three measures. We demonstrated a statistically significant crossed interaction between the side of H and the age at H with pragmatic language impairments [F_(1,36)_ = 17.48; *P =* 0.0002) and disorders in executive function (F(1,36) = 5.80; *P =* 0.021) in left early H and in right late H patients.	These results emphasize for the first time that hemispherotomized children have pragmatic language impairments that are independent of receptive language.	39	12,8 (2, 6)	Epilepsy Behav (2.96)
Howlin et al. (2014)	Cognitive and language skills in adults with autism: a 40-year follow-up.	To assess cognitive and language change in a cohort of individuals first diagnosed as children and followed up to mid-later adulthood.	For the majority of participants (N = 45, 75%), who were testable both as children and adults, IQ remained very stable and language also improved over time	For most individuals with autism who had an IQ in the average range (i.e. ≥ 70) as children, childhood IQ proved a reliable predictor of cognitive functioning well into mid- to- later adulthood.	60	2–13 (6)	Journal Child Psychol Psychiatry (2.21)
Höglund Carlsson et al. (2013)	Coexisting disorders and problems in preschool children with autism spectrum disorders.	To analyze cooccurring disorders and problems in a representative group of 198 preschool children with autism spectrum disorders (ASD) who had had interventions at a specialized habilitation center.	Children with autism had a mean of 3.2 coexisting disorders or problems, the ALC/Asperger group had a mean of 1.6, and children with autistic traits had a mean of 1.6. The most common disorder/problems in the total group pertained to language problems (78%), intellectual disability (ID) (49%), below average motor function (37%), and severe hyperactivity/ADHD (33%).	The results accord with the concept of early symptomatic syndromes eliciting neurodevelopmental clinical examination (ESSENCE) and highlight the need of considering ASD in a broad perspective taking also other cooccurring developmental disorders into account.	198	4, 5 – 6, 5 (2)	Scientific World Journal (2.10)
**Cognitive profile in participants with ASD and epilepsy**							
Villeneuve et al. (2014)	Cognitive and adaptive evaluation of 21 consecutive patients with Dravet syndrome.	To assess the cognitive and adaptive profiles of school-aged patients with Dravet syndrome (DS), we proposed to evaluate the intelligence and adaptive scores in twenty-one 6- to 10-year-old patients with DS followed in our institution between 1997 and 2013.	They did not find any significant correlation between the IQ or developmental quotient assessed between 6 and 10 years of age and the quantitative and qualitative parameters of epilepsy during the first 2 years of life in this small group of patients.	Despite an overall moderate cognitive deficit in this group of patients, the Vineland Adaptive Behavioral Scales described an adaptive/behavioral profile with low communication and autonomy capacities, whereas the socialization skills were more preserved.	21	6–10 (2,3)	Epilepsy Behav (2.96)
Ewen et al. (2019)	Epilepsy and Autism Severity: A Study of 6,975 Children.	To examine the extent to which ID, language dysfunction, and core autism symptoms mediate the relationship between ASD and epilepsy, and to assess a specific link between developmental regression and epilepsy in children with ASD.	They found that each severity factor-presence of intellectual disability, presence of language atypicalities, ASD-specific symptoms severity, and presence of motor issues-independently predicted a small increased risk for epilepsy, countering the argument that IQ alone is a risk factor.	Although severe epilepsy syndromes such as Landau-Kleffner syndrome are known to cause autistic-like symptoms following developmental regression, there is controversy about whether other forms of epilepsy are associated with the more common developmental regression seen in many young children with epilepsy.	6,975	6–17.9 (3.21)	Autism research (4.63)
Eom et al. (2014)	Routine developmental, autism, behavioral, and psychological screening in epilepsy care settings.	To assess the value of this screening and its validity for determining previously unidentified (‘actionable') problems in children with epilepsy.	Positive findings by test were 82% (ASQ), 54% (mCHAT), 15%, (SCQ), and 58% (SDQ). Findings were actionable in 46 children (20%): 18% of findings in children with established epilepsy and 23% of findings in patients with new-onset epilepsy. Of the 46 children for whom further referrals were made, the parents of 28 (61%) have pursued further evaluations.	Children with existing and new-onset diagnoses of epilepsy had actionable screening findings. These findings support the development of systematic screening of comorbidities for children with epilepsy.	236	4–6 (1)	Developmental Medicine and Child Neurology (2.77)

Lastly, with regards to the type of methodological design of the selected studies, quasi-experimental studies were conducted with evaluations at a single point in time, in a cross-sectional intra-group and inter-group manner, to assess the differences.

## Results

The selection of articles reviewed below follow the previously established inclusion and exclusion criteria, in addition to being the most recent articles, with the greatest relevance and sample of participants published to date. These are reported in thematic blocks according to the proposed objectives.

### The clinical characteristics of the user with ASD and epilepsy

An overview of the clinical characteristics and comorbidities of persons aged 5–15 years with epilepsy who, in turn, meet the diagnostic criteria for ASD is presented in the article by Reilly et al. (2015). In this investigation, 61% of users with epilepsy and ASD had a conduct disorder; a level similar to that of the general population with ASD. In those participants who met DSM-5 diagnostic criteria, 33% had hyperactive behavior and another 33% had linguistic and cognitive disturbances. The remaining third had no relevant data. It should be noted that hyperactivity features are common in children with ASD, especially in preschool children. Therefore, although there is a high rate of behavioral and psychiatric comorbidity in users with epilepsy and ASD, no significant difference was observed with the group of people with ASD without epilepsy compared with the group of children with ASD and epilepsy. These data follow the same trend as those obtained by functional neuroimaging techniques. For example, the research by Sharma et al. (2021) demonstrated the presence of specific EEG alterations in children with epilepsy and ASD, and further confirmed that these techniques allowed the identification of almost one fifth of children with ASD.

In another study carried out by Berg et al. (2011) a more specific range of ages of onset of diagnosis was included, specifically between 5 and 9 years. The results showed that 3.71% of the infantile population presented ASD and epilepsy in a comorbid manner. The study highlights that adolescents with childhood-onset epilepsy frequently have other disorders, such as hyperactivity, disruptive behavior, or cognitive or language difficulties. However, it is worth mentioning that no information was provided on the nature of the epilepsy in the participants and the characteristics that distinguished users with ASD from those without. Another interesting study regarding autism, epilepsy and age is the one performed by Viscidi et al. (2013). In it the authors state that in people with ASD two major peaks of epilepsy prevalence appear according to age. The first occurs in childhood, which corresponds to what was mentioned above. However, these authors state that there is another large peak around 12–14 years of age, since adolescents with the potential to develop epilepsy have more time to develop during their evolutionary course, something that is corroborated by studies such as that of Tuchman and Cuccaro (2011).

Age is a primary factor in the diagnosis of epilepsy, as there are works, such as that of Sathyabama (2019), showing that early recognition and initiation of early intervention result in better prevalence and improvement of symptoms. This study analyzed the clinical and symptomatologic characteristics of the ASD population in a sample aged 6 to 9 years. The results showed that 46.1% of the children with ASD had a comorbid psychological or medical diagnosis while 10.1% of them had epilepsy. The frequency of comorbid hyperactivity symptoms was 14.2%, while medical disorders were 15.1%, making a total of 29.3%. It was also indicated that children with ASD with less severity in their symptoms are diagnosed at relatively late ages, between 7 and 10 years, something that is also corroborated by other studies such as Mannion and Leader (2014b).

Other interesting results related to the general characteristics of the user with ASD and epilepsy are those of the study by Jokiranta et al. (2014), where three main findings stand out. Firstly, epilepsy was significantly associated with ASD and showed ten times higher odds of appearing together with ASD. Within this association, intellectual disability (ID) appeared as a predictor of altered cognitive functioning in children with ASD and epilepsy. This is because the presence of ID produces a linguistic and cognitive performance well below what is expected for their age. Secondly, effective treatment of comorbidity between ASD and epilepsy requires a thorough evaluation and screening to establish a differential diagnosis matched with correct pharmacological treatment. This is because in the study by Jokiranta et al. (2014), the authors confirm that all epileptic syndromes present a very similar concurrence with ASD, i.e., there is none that appears more frequently. Consequently, all syndromes must be considered when identifying them. This can make differential diagnosis very difficult, since it is easy for symptoms such as stereotyped behaviors or repetitive movements associated with ASD to be confused with epileptic symptoms, or *vice versa*. Thirdly, although the data indicate that the greatest risk of epilepsy occurs in early childhood, before the age of 3 years, the age of onset of epilepsy was not a distinguishing variable between users with and without ASD.

An interesting clinical feature in these disorders is the presence of disruptive behaviors. Research conducted by Norrelgen et al. (2015) showed that, in people with ASD and epilepsy, there was a higher prevalence of this type of behavior in the home environment than in the academic environment due to language and communication difficulties. A priori, it could be assumed that a greater number of cases and severity would be found in ASD residing in institutions or special education centers where children with greater linguistic and cognitive compromises go to school. However, significant differences were found in favor of the student assessment scores obtained by parents and teachers in regular schools, as opposed to those recorded by teachers in special education schools. According to Norrelgen et al. (2015), this is because in the context of the special education school, the child with ASD lives with other students with disorders or pathologies of equal or greater severity and, therefore, the characteristics of this may go more unnoticed or are addressed more efficiently. On the other hand, in the home context, parents may perceive more and worse disruptive behaviors or have more problems to address them.

Finally, regarding quality of life, in Celik et al. (2021) study on quality of life in ASD, the records of 154 patients were retrospectively analyzed to investigate the effect of the epilepsy diagnosis and factors related to how they perceived their life and the consequences of their children's diagnosis using the Quality of Life in Autism Questionnaire (Eapen et al., 2014). When an analysis of differences in quality of life among the participants in this study was conducted, a significant difference was appreciated between the mothers of 19 children with ASD-epilepsy and those of 30 children with ASD. Mothers of children with a combination of epilepsy and autism had a lower overall perceived quality of life and greater autism-specific difficulties compared to mothers of children with autism alone.

### Comparison between users with ASD and epilepsy with different sexes

The first included study referring to the sex of the patients was that of Amiet et al. (2013). In this work it was found that the prevalence of epilepsy was 41.2% in individuals with autism and intellectual disability vs. 11.1% in individuals with autism and normal intelligence, and that the worse the level of adaptive behavior the higher the prevalence of epilepsy. With regards to sex, the authors found that, in general, the prevalence of ASD was higher in males, but when the factors of epilepsy and intelligence quotient (IQ) are introduced, female sex appears as a significant variable. That is, the risk of epilepsy in people with ASD was significantly associated with intellectual disability in women, and that these differences are independent of IQ. For these authors, there are two risk and severity factors for epilepsy in autism: intellectual disability and female sex. In another study by Sharma et al. (2021), similar results were found, showing that the proportion of epilepsy in girls (24%) with ASD was higher than in boys (11%). In addition, these authors observed language and cognitive difficulties (82%) and a lower-than-normal IQ (88.6%) in most of the girls with ASD, which led them to state that this greater intellectual and motor disability in girls could be a consequence of the comorbidity between epilepsy and ASD.

A third study by Thomas et al. (2017), again, found significant differences in demographic and clinical characteristics in users with ASD and epilepsy with regards to sex. Overall, it was observed that there was a sevenfold increased risk of epilepsy in ASD compared to the general population without this disorder (8.6 vs. 1.2%). A higher prevalence of ASD was found in boys than in girls, but the latter had a higher risk of comorbidity with epilepsy, corroborating the data of Bolton et al. (2011). A higher prevalence of comorbid epilepsy was also seen in the population up to 17 years of age, as well as higher rates of intellectual disability and concurrent speech problems. Finally, epilepsy was more common in children with ASD with lower family income, consistent with what has been observed in the general population without ASD.

Finally, it is worth mentioning the work of Viscidi et al. (2013), whose results, once again, seem to confirm that there is a higher proportion of girls with ASD who have a low cognitive profile, together with an intellectual disability, which correlates with the presence of epilepsy. Another interesting finding, with respect to prevalence, is the existence of a greater number of children with ASD with epilepsy between the ages of 3 and 6 years, since the manifestations of epilepsy did not become evident until that age. This clinical profile was similar in both boys and girls, so no significant differences were found according to sex.

### The linguistic profile of the user with ASD and epilepsy

In the study by Norrelgen et al. (2015), a sample of 4- and 5-year-old children with a diagnosis of ASD or ASD and epilepsy who had not developed sentence utterance and structuring was selected. The data showed that approximately one in four children with ASD did not exhibit verbal language and that children who exhibited correct sentence production had a higher mean age than the group that did not exhibit non-verbal language. The observed age differences, according to the authors, could indicate that children with ASD acquire sentence production during the later preschool years before starting the primary school cycle. However, children with ASD and epilepsy would not acquire oral language production until later years, approximately after the age of 4 and 5 years, and may not even present oral language during the entire infantile stage. The results also showed that children with ASD and epilepsy present a greater delay in the acquisition of oral communication, compared to those children who do not have epilepsy.

Regarding the linguistic abilities of these users, the study by Save-Pédebos et al. (2016), analyzed the role of the left and right hemispheres in pragmatic skills in people with ASD and epilepsy. In this work, they show how children with right hemisphere damage develop an altered cognitive-linguistic profile from the early language stage, characterized by normal verbal reasoning, but with limitations in phonological, grammatical, and syntactic skills. Along with these alterations, the authors found a significant reduction in their pragmatics, with the way children use language to relate to their immediate environment being significantly affected. These data have been corroborated by Howlin et al. (2014), who studied comorbidity with language disorders in childhood patients with ASD and epilepsy. The rate of occurrence of language problems in these patients was very high (88.8%), although language and communication impairments may vary depending on the different degrees of ASD or type of epilepsy. For these authors, the imbalance in language development in children with ASD is the main symptom indicating a greater degradation of the individual's maturational development.

Finally, it is worth mentioning the work of Höglund Carlsson et al. (2013), which not only shows a high prevalence (56%) of language disorders in children with ASD and epilepsy, but also that this comorbidity correlates significantly with other areas, such as learning or physical development. For these authors, language impairment will negatively influence cognitive development and the degree of autonomy, needs and support of the person with ASD and epilepsy.

### The cognitive profile of the user with ASD and epilepsy

Regarding the cognitive profile of ASD children with and without epilepsy, in the study by Villeneuve et al. (2014), an analysis of the cognitive performance of individuals with ASD between 6 and 10 years of age was carried out to assess a global cognitive evaluation using the WISC-V (Wechsler, 2015). The highest rate of comorbidity was established with language alterations at 78%, followed by cognitive alterations at 44%. The authors did not find severe cognitive alterations at early ages, which they justified could be due to the fact that in that age range the requirements of the environment do not yet condition the appearance of this type of limitations. For them, it is in the school stage, which is much more demanding, where this gap in the acquisition of basic concepts and learning appears in comparison with the normative group of the same age. This could be one reason why children with ASD often do not see a neurologist until later ages, which in turn can lead to biases, delays, and errors in diagnosis, even more so when there are frequent comorbidities with ASD or intellectual disability (ID). In fact, it is essential to establish as soon as possible what the patient's symptoms are, since as the research by Viscidi et al. (2013) highlights, ID and ASD symptoms contribute independently to the risk of epilepsy in children with ASD. In this regard, the risk of epilepsy in children with ASD is not comprehensively explained by ID, which can complicate making an accurate differential diagnosis.

On the other hand, a factor that greatly affects the cognitive development and autonomy of people with ASD and epilepsy are disruptive behaviors, as they affect all the patient's contexts, impair their adaptability to the environment and negatively impact all activities of daily living. Studies such as Norrelgen et al. (2015) or Ewen et al. (2019) show that DI is directly associated with the presence of epilepsy, increased ASD symptoms and disruptive behaviors, and that children with ASD and epilepsy are at risk of presenting disruptive behaviors more frequently due to the higher probability of having a low IQ.

Finally, it is important to mention the study by Eom et al. (2014), which shows the enormous difficulty in making a correct assessment and diagnosis of ASD and epilepsy and their cognitive competencies, since it requires a detailed exploration and interpretation of the neurological and developmental status of the child. However, this work shows that the protocols of routine and screening evaluations of these users that are carried out in primary care centers are sensitive for the detection of cognitive alterations. However, to acquire greater reliability and validity in the assessment of the cognitive profile of these users, it is necessary to perform the examinations throughout the maturational process. For these authors, the use of tests of longer duration and complexity are not as useful as screening tests, either because of problems such as the time that can be dedicated to the patient, the context in which they are applied or the individual and evolutionary characteristics of the child.

For all these reasons, this review once again emphasizes the importance of an accurate differential diagnosis, in which all the clinical, cognitive-linguistic, and contextual characteristics of subjects with ASD and epilepsy are considered, emphasizing the need for an exhaustive evaluation of the characteristics of this population.

## Discussion

The main objective of this systematic review was to analyze the available evidence on the co-existence and relationship of epilepsy in the deterioration of cognitive-linguistic skills of those users who present an ASD in infancy. Likewise, the specific objectives were to analyze the general symptomatology of children with an ASD and epilepsy, and to explore the existing differences between both sexes in children with this comorbidity.

Regarding the general symptomatology of these comorbid disorders, the scientific literature has shown that, in neurological conditions in the pediatric setting, the impact on cognition and language exceeds the expectations of focal epilepsy and affects the function of multiple areas (Ortiz et al., 2009; Olmos-Hernández et al., 2013; Acquesta et al., 2018). However, research in this field is very limited due, in part, to the scarcity of studies, the small number of homogeneous samples, the variability of the manifestations of neurological alterations in clinical variables and their etiology; making studies inconclusive (Braakman et al., 2015; van Iterson et al., 2015; Specchio and Curatolo, 2021). So far, although changes in child development are an important factor which negatively influences the prognosis of children with epilepsy, none of the selected studies have specifically analyzed the effects of epilepsy and ASD comorbidity, their overall development, and their relationship with cognitive-linguistic competencies in the infantile population and gender in this population.

Regarding the differences with regards to age and sex, the reviewed articles show that if we observe the age of the participants in the studies, we will find that late diagnoses are milder precisely because the symptoms are less limiting. Therefore, many profiles with this comorbidity go unnoticed until later ages, when the demands of the environment will be more demanding and, therefore, will show the limitations of people with ASD and epilepsy. Likewise, in the female sex there was a higher prevalence than in the male sex with regards to the comorbidity of ASD and epilepsy. In addition, girls with this comorbidity show worse intellectual performance, presenting with ID in a high percentage of participants in the selected studies (Amiet et al., 2013; Thomas et al., 2017; Sharma et al., 2021). However, girls with ASD and epilepsy exhibit greater deficits in the language and cognition section (Thomas et al., 2017). It can be concluded that this symptomatologic profile could be due to the development of epilepsy, which is more pronounced in girls than in boys, especially at early ages, and that it causes minors to present a significant alteration in intellectual capacity which negatively affects quality of life and overall child development (Viscidi et al., 2013).

Regarding cognitive changes in pediatric epilepsy, it should be noted that they are part of the current definition of this comorbidity and have a significant impact on the adaptation of children to the environment, on the relationship with peers and on cognitive-linguistic development. Thus, they determine a poor prognosis and a poor quality of life for both the user and the entire family environment around him/her. In the last decade, the negative impact of early epilepsy on cognitive ability has been confirmed, establishing itself as a powerful predictor of difficulties in the development of these functions in children with ASD (Karrasch et al., 2017) and causing all areas of language and cognition to show a reduced performance. When their impact is analyzed in relation to other clinical variables such as duration, frequency, or time of development, the early onset of this disorder is the variable that presents the greatest predictive weight in the production of these alterations. This may be due to the fact that, in childhood, the mechanisms of neuronal plasticity are at their peak, trying to consolidate the mechanisms of learning and memory through excitatory processes (Jensen, 2011). However, an excess of neuronal activation due to epileptic episodes can have a cascading effect that impairs the entire subsequent cognitive development of children. Previous studies have also shown that all these factors correlate negatively with IQ (Lee et al., 2014) and cognition (Riva et al., 2005; Campiglia et al., 2014). Seizures in the first year of life are associated with greater impairment in intelligence level (Campiglia et al., 2014), and may cause metabolic or structural abnormalities, which may later affect cognitive development (Braakman et al., 2012; Lee et al., 2014). In ASD, cognitive conditions are particularly sensitive to the early onset of epilepsy (Malcher-Lopes, 2014), which supports the early vulnerability hypothesis stating that seizures in the first year of life are associated with greater impairment in intelligence level (Campiglia et al., 2014). This hypothesis alludes to the period in which a minor presents a high risk of developing alterations in their evolutionary milestones that would imply a considerable deterioration in their motor, adaptive, communicative, cognitive, or social abilities. In this regard, it has been shown that the age span of up to 3 years old could be the most vulnerable critical point, especially in terms of memory and cognitive flexibility (Anderson et al., 2010).

Regarding language skills, the performance of children with ASD and epilepsy appears to be lower than that found in children with ASD alone (Höglund Carlsson et al., 2013; Sharma et al., 2021). This may indicate that, in the case of epilepsy, the language skills assessed may be significantly impaired (MacAllister and Schaffer, 2007); mainly those dealing with oral language utterances. This could be explained by the alteration of different functional areas in charge of language production, highlighting pragmatics as the most notorious dimension. However, other aspects such as semantics, morphosyntax and phonology can also exhibit slight difficulties (Braakman et al., 2015). Syntactic structure is also altered, since children with ASD and epilepsy produce errors when organizing the elements of a sentence with a correct grammatical structure (Save-Pédebos et al., 2016). Regarding the location of the alterations, functional neuroimaging studies with EEG have shown that the activation of the multimodal cortex during semantic processing is supported by the ventral network connecting the frontal lobe and temporal lobe through the inferior occipital frontal lobe. This implies that people with ASD and epilepsy show alterations in lexical acquisition, as well as in the production of oral discourse in accordance with their maturational age (Friederici and Gierhan, 2013). Furthermore, the proposed results may explain the lack of significant differences between the left and right hemispheres, especially in language skills. This is due to differences with adults, as the left hemisphere language neural network of children is not as significantly lateralized. All this shows that activation contrasts with the contralateral cortex during the process of oral communication (Save-Pédebos et al., 2016).

Finally, it should be noted that there is a lack of scientific literature that addresses this issue directly and obtains significant data. At present, only a few studies address the linguistic and cognitive characteristics of children with ASD and epilepsy, so this systematic review has only been able to select a total of 18 articles that meet the required quality criteria. In this sense, there is no research in the current scientific literature that shows a comparison between cognitive and linguistic skills in children with comorbidity between ASD and epilepsy. All this leads us to conclude that the present systematic review has a useful value for the field of research with ASD, being a novel study that provides accurate and precise information on a current problem. This may open the door to new lines of research that relate the linguistic-cognitive aspects of users with ASD and epilepsy, which highlights the need to focus on this topic. This is imperative, since in the coming years a considerable increase in profiles with these characteristics and conditions is expected due, in part, to poor nutrition, exposure to narcotic substances during gestation, obesity, existence of a psychiatric disorder, advanced age or autoimmune diseases, stress, and infections of the mother (Arroyo, 2022). Therefore, relevant information should be provided to the professionals who will oversee the enhancement of the skills of this population, as well as improving their quality of life.

## Data availability statement

The raw data supporting the conclusions of this article will be made available by the authors, without undue reservation.

## Author contributions

AC-V, ML-Z, and FM-F conceptualized the study. AC-V wrote the review protocol and original draft of this manuscript, which was reviewed by ML-Z and FM-F. AC-V and ML-Z conducted the database search. AC-V and FM-F screened the studies and extracted the data. FM-F acted as a third reviewer and resolving disagreements. All authors contributed to the article and approved the submitted version.
